# Remodeling of Retinal Architecture in Diabetic Retinopathy: Disruption of Ocular Physiology and Visual Functions by Inflammatory Gene Products and Pyroptosis

**DOI:** 10.3389/fphys.2018.01268

**Published:** 2018-09-05

**Authors:** Rubens P. Homme, Mahavir Singh, Avisek Majumder, Akash K. George, Kavya Nair, Harpal S. Sandhu, Neetu Tyagi, David Lominadze, Suresh C Tyagi

**Affiliations:** ^1^Eye and Vision Science Laboratory, Department of Physiology, University of Louisville School of Medicine, Louisville, KY, United States; ^2^Department of Physiology, University of Louisville School of Medicine, Louisville, KY, United States; ^3^Department of Biochemistry and Molecular Genetics, University of Louisville School of Medicine, Louisville, KY, United States; ^4^Department of Ophthalmology and Visual Sciences, University of Louisville School of Medicine, Louisville, KY, United States; ^5^Kentucky Lions Eye Center, University of Louisville School of Medicine, Louisville, KY, United States

**Keywords:** chemokines, cytokines, diabetic retinopathy, epigenomics, homocysteine, inflammation, pyroptosis, signaling pathways

## Abstract

Diabetic patients suffer from a host of physiological abnormalities beyond just those of glucose metabolism. These abnormalities often lead to systemic inflammation via modulation of several inflammation-related genes, their respective gene products, homocysteine metabolism, and pyroptosis. The very nature of this homeostatic disruption re-sets the overall physiology of diabetics via upregulation of immune responses, enhanced retinal neovascularization, upregulation of epigenetic events, and disturbances in cells’ redox regulatory system. This altered pathophysiological milieu can lead to the development of diabetic retinopathy (DR), a debilitating vision-threatening eye condition with microvascular complications. DR is the most prevalent cause of irreversible blindness in the working-age adults throughout the world as it can lead to severe structural and functional remodeling of the retina, decreasing vision and thus diminishing the quality of life. In this manuscript, we attempt to summarize recent developments and new insights to explore the very nature of this intertwined crosstalk between components of the immune system and their metabolic orchestrations to elucidate the pathophysiology of DR. Understanding the multifaceted nature of the cellular and molecular factors that are involved in DR could reveal new targets for effective diagnostics, therapeutics, prognostics, preventive tools, and finally strategies to combat the development and progression of DR in susceptible subjects.

## Introduction

Despite advances in medical health and technologies, the incidence of diabetes has reached epidemic proportions globally and thus diabetic complications are increasing throughout the world, e.g., DR. According to WHO, the diabetic population has risen by 314 million between 1980 and 2014 (Mathers and Loncar, 2006) and the CDC projects that Americans with diabetes will at least double or even triple in 2050, meaning one in every three adults in the United States would be affected if the current trend continues unabated. It is hard to imagine what this steep increase might have on the overall health care system in United States and the world since the prevalence of diabetes-related complications will keep rising proportionally in the affected patient populations (Mattei et al., 2015). In short, WHO believes that diabetes is an epidemic and therefore it may lead to a major health crisis in future. DM is divided into two main categories: T1D and T2D. T1D is characterized by a total or near total loss of insulin production, and thus patients are insulin-dependent. In contrast, T2D is characterized by insensitivity to insulin, and the patients can be treated with other drugs along with insulin during the early phases of the disease (Ferris et al., 1999; Nathan, 2015). Diabetes complications generally group into microvascular or macrovascular complications. Microvascular disorders include retinopathy, nephropathy, and neuropathy while macrovascular include coronary artery disease, stroke, and other peripheral vascular disease (Barrett et al., 2017; Huang et al., 2017). If these pathologies are not managed appropriately, then significant vascular and thus end-organ damage can occur, including the loss of retinal architecture. Eventually, new blood vessels (NV) will grow profusely superficial to and inside the retina leading to devastating outcomes in the untreated patients.

Diabetes can affect almost every part of our visual system, but vision loss and blindness occur predominantly because of retinal complications if timely and effective interventions are not sought by the patients (Gori et al., 2005). DR has already become a leading cause of acquired blindness in the United States and other developed nations. Unfortunately, ∼2.6% of global blindness cases can be attributed directly to diabetes (Bourne et al., 2013). Approximately 40% of diabetics over 40 years of age have some form of retinopathy; of which 8.2% exhibit visual symptoms and vision related complications (Ciulla et al., 2003; Klein et al., 2008). Landmark clinical trials established that hyperglycemia is a critical factor in the development of DR (Nathan, 2014). Thus, identifying early pathogenetic events in the disease process may provide useful insights that could lead to the identification and deployment of appropriate diagnostics and therapeutic targets for preventing the serious micro- or macrovascular complications that could arise later, particularly retinopathy (van Wijngaarden et al., 2005; Gross and Glassman, 2016).

Hyperglycemia’s harmful effects are directly related to the structural damage inflicted on small retinal blood vessels and are governed by the systemic and local ocular factors. Although there are genetic factors also at play, but individuals with elevated blood glucose levels have severe retinopathy than those with strict blood glucose control (Kuo et al., 2014; Pradhan et al., 2016). HbA1c measures overall glucose level in blood over a period of 3 months. HbA1c levels less than 7.0% are generally recommended to minimize the risk of vascular complications, including DR. On the other hand, increased levels of blood lipids may also impart some role in the progression of DR as lipid-lowering compounds such as fenofibrate offers benefits in preventing the progression of DR. The complete details of the mechanism(s) underlying this protection are not well understood (Knickelbein et al., 2016; Ju et al., 2017; Liu et al., 2017). Moreover, the genetics and epigenetics of an individual can influence susceptibility to DR and response to the treatment. However, the exact number and nature of the genes involved or the epigenomics elements that are involved remain elusive. Other demographic risk factors for DR include Hispanic or African American ethnicity, systemic hypertension, duration of disease, and pregnancy (Gupta and Misra, 2016; Penman et al., 2016; Varma et al., 2016; Davidson, 2017; Jin et al., 2017; Kowluru, 2017; Mastropasqua et al., 2017; Zhang et al., 2017). DME is one of the most common complications and the cause of vision loss in diabetics. The early stage of DR is called non-proliferative diabetic retinopathy (NPDR) and is characterized by the presence of intra-retinal hemorrhages, microaneurysms, intra-retinal microvascular abnormalities, and cotton wool spots (fluffy white patches in the retina caused by focal swelling in the retinal nerve fiber layer). Development of retinal NV is called PDR, an advanced form of DR that carries a risk of other structural complications. Retinal changes occur after approximately a decade of living with T1D. One study reported nearly a 50% chance of developing PDR after 25 years of diabetes. Recently, progression to PDR and severe visual loss has become less, reflecting a strict control of hyperglycemia, serum lipids, blood pressure, and earlier diagnosis (Scanlon et al., 2013).

There are three principal ways by which DR can cause vision loss: (1) vitreous hemorrhage or TRD due to PDR (Crawford et al., 2009), (2) DME (Wilkinson et al., 2003; Lee et al., 2015), and (3) macular ischemia (Sim et al., 2013). In PDR, the new vessels are incompetent and can easily hemorrhage into the pre-retinal space or vitreous cavity, causing severe vision loss, often instantaneously. The vessels ultimately become fibrotic if left untreated and can exert traction on the retina, causing a TRD phenotype. DME involves fluid leakage out of retinal capillaries into the central retina (macula), the visually significant portion of the retina. Finally, macular ischemia, as the name implies, involves a critical lack of perfusion to the macula due to microvascular complications, causing dysfunction and even death of retinal cells (Hardy et al., 2005; Simo et al., 2006; Wong et al., 2016). Without therapeutic intervention, DR will result into the vision loss or blindness.

Management of DR starts with screening patients for the signs of retinopathy and then treating them if vision-threatening lesions are identified. Typically, DME is treated with intravitreal administration of anti-VEGF based biologics or small organic molecules such as steroids (Hodzic-Hadzibegovic et al., 2017; Koyanagi et al., 2018). Anti-VEGF agents have primarily replaced focal or grid laser photocoagulation of the macula as the first-line treatment of DME, although this remains an important adjunct therapy in refractory cases. In practice, the management of DR aims at preventing or delaying the onset of retinopathy by controlling high blood pressure, blood sugar, and lipids levels. Introduced approximately 60 years back, photocoagulation is still an effective treatment for PDR (Evans et al., 2014). Recent clinical trials have established that PDR can also be treated with repeated anti-VEGF injections, though it is still unclear how long a patient be treated if one opts for the injection strategy. Other drugs that are currently being tried are the potential agonists for peroxisome proliferator activated receptors (PPARs), plant extracts such as forskolin (it binds to glucose receptor, specifically GLUT1), minocycline (it serves as an anti-inflammatory agent), celecoxib, another COX-2, and angiopoietin 2 antagonists. Anti-VEGF agents have been shown to have a disease-modifying effect (Ip et al., 2015). However, such an approach carries a significant treatment burden (Blanco-Garavito et al., 2017; Reich et al., 2017; Wong et al., 2017). Thus, there remains a considerable unmet medical need for the development of effective strategy (ies) to prevent DR or at least slow its progression. Critical aspects of this endeavor are to ensure that the anticipated intervention is safe, well tolerated, and less burdensome than the currently available options. Some investigations have implicated inflammation and pyroptosis as a possible cell-molecular mechanism in DR biology (Feenstra et al., 2013; Volpe et al., 2016).

## Inflammation and Pyroptosis Disrupt Physiological Homeostasis in the Eye

**FIGURE 1:**
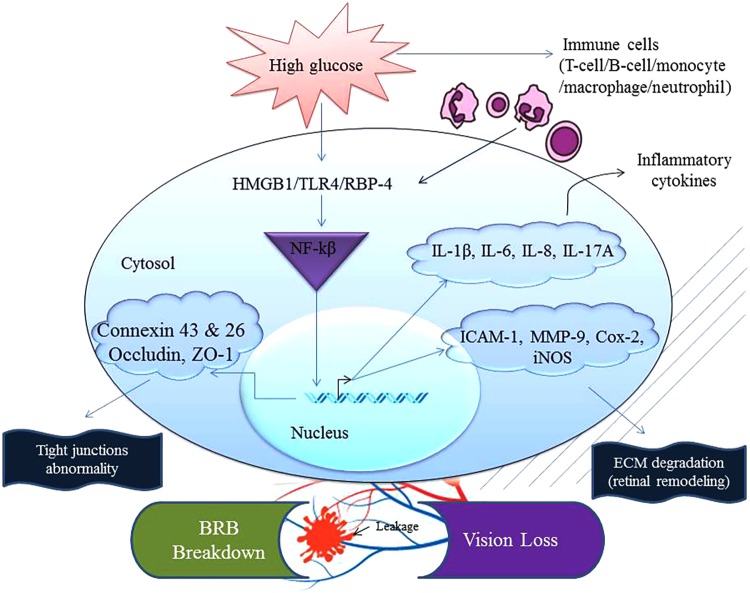
Chronic exposure to high glucose and the recognition/sensing of a danger signal from concomitant infection(s) can accelerate inflammation. Abnormal physiological homeostasis is common in the ocular compartment of a patient suffering from diabetes. This augmented nature of inflammation increases expression of mediators that can interact together to activate NF-κB inducing transcription of inflammatory factors such as TNF, iNOS, IL-1β, IL-6, IL-8, IL-17A, adhesion molecule (ICAM-1), eicosanoids (Cox-2) and extracellular matrix degradation enzyme (MMP-9). These events end up compromising the cellular tight junctions and thus communication between ocular cells leading to blood-retinal barrier (BRB) breakdown affecting occludin, ZO-1, connexin 43 and 26. Cox-2, cyclooxygenase-2; HMGB1, high-mobility group box 1; iNOS, inducible nitric oxide synthase; IL-1β, interleukin-1 beta; ICAM-1, intercellular adhesion molecule-1; MMP-9, metalloproteinase-9; TLR4, toll-like receptor 4; TNF, tumor necrosis factor; RBP-4, retinol binding protein-4; ZO-1, zona occludin-1.

During the pathogenesis of DR, inflammatory mediators are elevated before any anatomical or histopathological alterations are seen because hyperglycemia induces metabolic dysregulation that causes cells to malfunction and give rise to a chronic state of low-level inflammation in the body. A concurrent infection and the subsequent mounting of an immune along with existing inflammation in diabetics can further activate additional pro-inflammatory pathways. These can potentially further disturb the homeostatic balance in the eyes (Joshi et al., 1999; Casqueiro et al., 2012; Kitagaki et al., 2016; Heller et al., 2017). This heightened immune reaction, in the long run, plays havoc with the host’s cellular functions. New evidence now indicates that retinal inflammation plays a dominant role in the pathogenesis of DR (Zhang et al., 2011; Whitcup et al., 2013; Ascaso et al., 2014). Data from experimental findings and clinical studies revealed a set of inflammatory cytokines, chemokines, and their receptors that were acutely expressed in the RPE, blood, vitreous, and aqueous humor of the patients. In other words, upregulation of the inflammatory mediators and cross-talk between them cause microvascular changes and breakdown of the BRB (**Figure 1**).

Homocysteine; a homolog of the amino acid cysteine has been implicated in a variety of thrombotic and vascular occlusive diseases via an array of inflammatory signatures. In a clinical study of plasma homocysteine from diabetics and controls, the mean Hcy level recorded was 11.75 ± 0.24 in the control group, 13.46 ± 0.74 in people with diabetes who had no DR, 14.56 ± 0.64 in subjects with NPDR, and 15.86 ± 1.34 in subjects with PDR. Thus, Hcy levels seem correlative with diabetes, and the severity of DR. In general, hyperhomocysteinemia (HHcy) is defined as a condition when Hcy levels are higher than the 15 μM/L. The data indicated that HHcy was significantly higher in the NPDR and PDR groups relative to the control group. These observations and other findings suggest that HHcy is associated with DR, and it can explain the increased risk of microvascular angiopathy in diabetics (Goldstein et al., 2004). Interestingly, Hcy is metabolized to homo-thiolactone (HCTL) which is an intra-molecular thioester of Hcy and is regarded as more toxic than Hcy. Paraoxonase (PON) exhibits esterase activity and lactonase activity (PON-HCTLase) that help detoxify HCTL. PDR patients show a significant increase in HCTL and PON-HCTLase activities and that have been confirmed in *in vitro* studies using the bovine retinal endothelial cells (RECs) depicting a direct proportional relationship in PON-HCTLase activity and mRNA expression levels of PON2 in a dose- and time-dependent manner when treated with Hcy and HCTL. It was concluded that elevations were probably a protective effect of eliminating HCTL, which mediated endothelial cell dysfunction. Furthermore, a bioinformatics analysis revealed that HHcy could modulate the structural and functional aspects of PON in PDR and could also affect the dual enzyme activity of PON. These findings indicate the possibility of PON activity and changes in HCTL expression as potential biomarkers for screening and detection of DR patients (Barathi et al., 2010). In our early study, we also demonstrated that Hcy could potentially mediate the expression of inflammatory markers (e.g., chemokines, cytokines, and interleukins receptor molecules) in human retinal cells without interfering with their cellular morphologies or their genomic integrities (Singh and Tyagi, 2017a).

Inflammation plays an essential role in the early pathogenesis of DR (Kern, 2007; Tang and Kern, 2011; Rangasamy et al., 2012; Kastelan et al., 2013). As the disease progresses, a corresponding increase in inflammatory factors along with leukocyte adhesion to inflamed endothelium affect the integrity of the BRB in diabetic eyes. As we know, maintenance and regulation of the BRB are required for normal retinal function. Loss of this barrier can result in the panoply of retinovascular diseases, including DR. In fact, loss of BRB integrity is associated with DR which can lead to vascular leakage and development of macular edema. In this context, DME can result in decreased vision and even blindness if patients fail to seek timely medical intervention due to leakage and blockage of micro-vessels (Bhagat et al., 2009). In diabetic patients and diabetic animal model studies, studies have shown an increase in expression of many pro-inflammatory mediators including TNF-α, IL-6, and IL-1β in both the vitreous and the retina. These inflammatory markers were associated with an increase in permeability in retinal vessels, particularly in diabetic eyes. Studies have also shown that hyperglycemia injures retinal vascular morphology through activation of a pro-inflammatory phenotype in endothelial cells that is characterized by the upregulation of cell surface adhesion molecules (e.g., ICAM-1, VCAM-1) (Kim et al., 1994; Morigi et al., 1998). These molecules, in turn, facilitate the adhesion and subsequently the transmigration of leukocytes across endothelial lines, leading to a retinovascular inflammatory hotbed, resulting in capillary occlusion, and apoptosis of the endothelial cell. These injuries eventually induce the breakdown of the BRB (Miyamoto et al., 1998, 1999; Miyahara et al., 2004). Therefore, an anti-inflammatory treatment could be very useful in preventing further damage from DR (**Figure 1**) (Joussen et al., 2001, 2003; Miyahara et al., 2004).

Sequence of events during the immune response that leads to BRB alterations and subsequent breakdown appears to be as follows: (a) upregulation of endothelial adhesion molecules [e.g., ICAM-1, VCAM-1, platelet and endothelial cell adhesion molecule 1 (PECAM-1)], (b) leukocyte adhesion to the endothelium, (c) simultaneous release of chemokines, cytokines, and vascular permeability factors, (d) decrease in tight-junction associated proteins, and finally (e) inability of white blood cells to penetrate the neuro-retinal parenchymal structures. Important pro-inflammatory chemokines and adhesion molecules contributing to the initiation and progression of DR phenotypes, include endothelin-1, Interleukins [interleukin 1β (IL-1β), interleukin 8 (IL-8), interleukin 1α (IL-1α), interleukin 6 (IL-6)], monocyte chemotactic protein 1 (MCP-1), p-selectin, and CXCL10/IFN induced protein 10 (IP-10). Additional cytokines that are also associated with DR are angiopoietin 2 (Ang 2), tumor necrosis factor alpha (TNF-α), platelet-derived growth factor AA (PDGF-AA), the members of VEGF family, and TGF-β. While a variety of chemokines are elevated in diabetic eyes, chemokine ligand 2 (CCL2) is more prevalent in both the vitreous and the serum (Elner et al., 1995; Dominguez et al., 2016). CCL2/MCP-1 influences vascular inflammation via activating and recruiting leukocytes during hyperglycemia in various retinal cells (e.g., pigmented epithelial cells, Muller’s glial cells, retinal vascular endothelial cells) (Bian et al., 2001). Furthermore, *CCL2* gene polymorphisms were found to be associated with the gradual development of DR in susceptible hosts (Maier et al., 2008; Dong et al., 2014; Urias et al., 2017). BRB breakdown was inhibited in a diabetic *CCL2* knock-out mice model, hence directly implicating this molecule. Thus, inhibition of *CCL2* may selectively prevent disruption of BRB in DR related pathologies (Rangasamy et al., 2014). By-products of glucose metabolism are transformed non-enzymatically into advanced glycation end-products (AGEs). AGEs can further react to make reactive species like dicarboxylic compounds. Furthermore, it was reported that AGEs tend to accumulate in the retina and cornea in diabetics indicating the onset of pathological alterations inside eyes. *In vivo* studies confirmed that exposure to AGEs increases VEGF expression along with inflammatory mediators such as ICAM-1 via nuclear factor-kappa beta (NF-κB) (Stitt, 2003; Alghadyan, 2011). As DR progresses, it causes significant thickening of the capillaries’ basement membrane in different compartments of the retina [e.g., the inner plexiform layer (IPL) relative to the outer plexiform layer (OPL)] (Anderson et al., 1995). DR can also lead to the induction of apoptosis in glial, ganglion, and Müller cells as well as in the capillary network of the retina (Kowluru et al., 2012).

It is becoming increasingly evident that MMPs are primary regulators of the innate as well as acquired immune networks in our body (Kessenbrock et al., 2010; Singh and Tyagi, 2017c), shedding light on the development of DR pathology (Parks et al., 2004). MMPs are primarily produced as zymogens; inactive enzymes which contain a pro-peptide domain that needs to be cleaved. The pro-peptide domain contains a cysteine residue that interacts with zinc in the enzyme-binding site, thus preventing enzyme activity. Some of the MMPs do have a prohormone convertase cleavage site, a furin-like entity, which when cleaved activates the enzyme. Some MMPs also include a transmembrane segment in their domains (Pei et al., 2000). Immune-related molecules such as CCL/monocyte chemoattractant family protein members are activated by MMP cleavage. MMPs and their regulators are upregulated in the diabetic vitreous (Tuuminen and Loukovaara, 2014). This observation was consistent in animal models of diabetes which showed the retinal upregulation of MMP-2 and MMP-9 (Giebel et al., 2005). Accordingly, MMP inhibitors have been shown to significantly decrease retinal vascular permeability and loss of junctional proteins in diabetic animals (Navaratna et al., 2007; Rangasamy et al., 2012). These observations suggest a plausible mechanism for BRB breakdown via the proteolytic degradation of VE-cadherin (Navaratna et al., 2007). In fact, hyperglycemia can activate many other types of soluble mediators such as AGEs and a range of potent inflammatory cytokines that can increase MMPs’ expression levels. Findings demonstrated that retinal inflammation could attract an increased number of leukocytes to the retina, as mentioned earlier, which in turn can bind to the vascular endothelium to activate cellular proteinases like elastase which in turn cleaves VE-cadherin and its associated proteins from the cell surface repertoire. Based on these observations, it appears that these proteinases may serve as potential therapeutic targets for DME (Allport et al., 2000).

Interestingly, remodeling of the retina and its altered architecture in DR can be explained by the active role played by a host of MMPs and other events like pyroptosis. As we know that MMPs are implicated in numerous cellular functions such as degradation of ECM, cell proliferation, apoptosis, corneal collagen formation, organ development as well as in the causation of internal inflammation. Some of these proteins like MMP-2 are most prevalent in cells and tissues while MMP-9 is quite complicated in its functionality. Notably, MMP-9 levels are different among various ethnic groups while there is no significant difference between genders (Tayebjee et al., 2005). MMPs are involved in the pathogenesis of DR since MMP-9 is activated through Ras/Raf/mitogen-activated protein MAPK/ERK pathway in the retinal and retinal capillary cells of diabetics. MMP-9 plays a role in the death of retinal capillary cells and that increases vascular leakage through disruption of the tight junction’s complex, and degradation of the tight junction protein occludin. They also participate in the process of DR NV (Kowluru et al., 2012). Studies show that MMP-9 participates in the development of DR via two routes: by inducing apoptosis in retinal capillaries cells during the early disease stage and NV in the later stage (Salzmann et al., 2000; Giebel et al., 2005; Kowluru and Zhong, 2011; Kowluru et al., 2011, 2012). MMPs also participate in a host of organ-specific (e.g., retinopathy, nephropathy, and cardiomyopathy) diabetic complications due to a high glucose environment that stimulates secretion of these zinc-dependent proteinases during disease progression. In fact, animal models that have been specifically developed and studied simulating DR phenotypes and when their retinal and vitreous samples were examined along with diabetic retinopathic patients they all showed a significant increase in the levels of MMP-9 and MMP-2 (Kowluru et al., 2012). Thus, hyperglycemia does affect the activity of MMPs in the eye, and they can certainly compromise the integrity of the BRB. Interestingly, physical activity such as exercise can ameliorate hyperglycemia as well as can lower the levels of MMPs, and therefore can potentially help prevent or at least slow down the pathogenesis of DR in susceptible individuals.

Evolutionarily, the eukaryotic cell is fully capable of starting several cell death programs when confronted with that option that may fall into two broader categories: non-inflammatory or pro-inflammatory. Cell death programs include necrosis, apoptosis, and pyroptosis (Sarks et al., 1988; Kaneko et al., 2011; Tarallo et al., 2012). Apoptosis is non-inflammatory while others induce inflammation and various cell death pathways can be identified by certain markers. Apoptosis is identified via the caspase while pyroptosis is identified by the inflammasome activity (Bergsbaken et al., 2009). Pyroptosis is inherently an inflammatory event in its origin and can be triggered by a host of stimuli including the CVDs such as stroke and heart attack, cancer, neurological disorders, etc. Pyroptosis is also crucial for controlling microbial infections, and it is known that the competition between the host and pathogen can modulate the process of pyroptosis. Pyroptosis signaling pathways that have been studied extensively employ the NLR family pyrin domain containing 3 (NLRP3) inflammasome. This pathway includes apoptosis-associated speck-like protein containing a carboxy-terminal CARD, caspase-1, bridging adaptor, and of course the NLRP3 and is activated when NF-κB is activated and that increases IL-1β and NLRP3 expression. It also triggers inflammasome assembly to generate IL-18 and IL-1β. Recent studies have identified pyroptosis in aged-related macular degeneration leading to the RPE cell death (Gao et al., 2015). As discussed above for DR, all these factors are present such as the activation of NF-κB, upregulation of IL-1β and IL-18. Other related studies have shown that diabetic animal models deficient in NLRP3 have attenuated DR signatures (Schroder et al., 2010). Additionally, some studies showed the presence of NLRP3 in the vitreous of PDR patients (Loukovaara et al., 2017). It is worth to point out that we are just beginning to understand the finer details and related molecular mechanism(s) that govern pyroptosis and other related processes which are downstream of the caspase-1 activation pathway in the eyes. In future, identification of potential markers may likely provide further insights that can allow us to comprehend this process in more details. However, it is important to remember that composition of the inflammasome may too play pathogenic role(s) and thus can determine the fate of the inflamed cells in the eyes that have been exposed to varied responses because of chronic stimuli environment. Thus, pyroptosis and inflammation are relevant to our understanding of their effects during serious medical conditions for which inflammation is central to the pathophysiology of diseases such as DR. In fact, DR shares many similarities with other inflammatory diseases and as a result it is being recognized now as the central player in DR via various mechanisms including pyroptosis (Joussen et al., 2001; Singh and Tyagi, 2017b).

## Retinal Neovascularization Leads to Blindness During Diabetic Retinopathy

**FIGURE 2:**
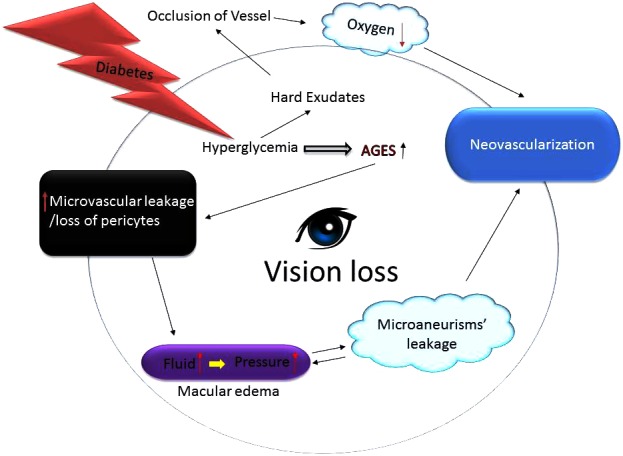
Growth of abnormal blood vessels in the eyes of diabetics lead to blindness. Hyperglycemic environment increases generation of the advance glycation end products (AGES) harming capillary pericytes and vessels’ permeability thereby spilling fluid contents into the surroundings. The fluid leakage builds up eye pressure that can burst micro-aneurysms. Since the blood vessels fail to deliver enough oxygen and nutrients the eyes upregulate compensatory mechanisms including neovascularization. Additionally, excess glucose also causes formation of hard exudates that can occlude vessels thus further impairing vessels’ ability to nourish the retina thereby leading to vision loss or blindness.

Similar to the pathology of the neovascular glaucoma (NVG); the slow growth of new blood vessels that occurs in the iris as well as the trabecular meshwork (the proverbial “drain” of the eye) inhibits outflow of the aqueous humor fluid. Consequently, intraocular pressure rises precipitously which can cause irreversible damage to the optic nerve leading to blindness if left untreated. Same way, the corneal epithelium in people with diabetes is prone to sloughing and epithelial erosions (e.g., breakdown of the epithelial surface or corneal abrasions) and that can develop into a chronically non-healing epithelial defect which is painful and that can decrease the vision (Bikbova et al., 2012; Vieira-Potter et al., 2016). Likewise, in early DR the patients may be asymptomatic which is clinically called as NPDR. The only cause of vision impairment in NPDR is DME, which is present only in a minority of cases with mild NPDR. However, as stated above, during the late stage of the disease, small, new blood vessels (NV) grow on top of the retina and into the pre-retinal space or the vitreous (**Figure 2**). As we know that the retinal vascular unit is mainly composed of endothelial cells, pericytes, and astrocytes. Diabetes over time changes the integrity and structure of this retinal neurovascular unit (Shin et al., 2014). The production of various growth factors by the inflamed retina may also trigger an inflammatory cascade that further drives NV. Unlike NPDR, PDR causes hemorrhage into the vitreous cavity, which frequently results in a sudden and profound loss of vision. On the other hand, the principal cause of retinal NV in PDR is hypoxia wherein VEGF is released from retinal cells in response to hypoxia. VEGF being an anti-apoptotic factor can promote endothelial cell division, vasodilation, and increase in vascular permeability (**Figure 2**). VEGF is also capable of activating PKC-β which phosphorylates the tight junction protein, occludin. The phosphorylated occludin is then ubiquitinated and targeted for degradation leading to an increased vascular permeability (Lai et al., 2005; Murakami et al., 2012; Manolov et al., 2014). VEGF is made by many types of retinal cells, including RPE, Muller, ganglion, glial, and endothelial cells (Huang et al., 2011; Sun et al., 2012) and it has been studied extensively. It is considered a critical factor related to the BRB breakdown and its levels have been found to be significantly higher in DME patients than non-diabetics ones (Funatsu et al., 2002; Caldwell et al., 2003). The biology of VEGF is unique in the sense that by serving as a vasoactive cytokine, it can also increase vascular permeability, thereby causing extravasation of fluid into the retinal space. VEGF can also efficiently phosphorylate important proteins, e.g., VE-cadherin, occludin, and ZO-1, leading to BRB impairments (Caldwell et al., 2003). Additionally, VEGF can stimulate leukostasis in small retinal vessels. These leukocytes further release more cytokines or migrate via the *trans*-endothelial routes, thus exacerbating BRB dysfunction in the eyes (**Figure 1**) (Aiello et al., 1994, 1997).

There are several other pathways beyond VEGF that are important in retinal NV and are plausible therapeutic targets. The one under active investigation is the angiopoietin-Tie2 signaling pathway. Angiopoietin 1 (Ang1) is a protein that acts as an agonist of Tie2, a receptive tyrosine kinase mostly expressed on endothelial cells. This ultimately results in the stabilization of the endothelium and inhibits retinal NV when VEGF levels are high. Conversely, angiopoietin 2 (Ang2) dephosphorylates Tie2 which destabilizes endothelial cells and thus promotes NV (Campochiaro, 2013). Vitreous levels of Ang-2 are significantly upregulated in patients with severe macular edema, thus indicating that Ang-2 may be associated with impairment of the BRB. Furthermore, in a study when Ang-2 was injected in the vitreous of non-diabetic rats, it was concluded that it significantly increased vascular permeability in the retina. Other studies found that Ang-2 contributed to VE-cadherin loss via phosphorylation (Patel et al., 2005; Rangasamy et al., 2011). Ang-2 can further increase inflammation via inducing sensitivity of endothelial cells to TNF-α, which then upregulates the expression of ICAM-1 thus promoting adhesion of monocytes. Moreover, VEGF and Ang2 act synergistically to cause NV, but interestingly Ang2 in the setting of low VEGF levels can actually inhibit the process. The synergy displayed by Ang2 and VEGF has prompted interest in combined VEGF and Ang2 inhibition. A phase II trial of a single monoclonal antibody that inhibits both targets was completed in early 2018 and showed additional efficacy over anti-VEGF therapy alone for DME (Dugel et al., 2018). This represents a promising new target both for achieving more rapid visual gains in DME and possibly in treating the subset of patients whose DME is non-responsive to anti-VEGF therapy. Since anti-VEGF therapies remain the treatment of choice for DME and are also a reasonable alternative treatment to PRP for the treatment of PDR. Despite some concerns about its heavy usage, clinical trials involving patients followed for 5 years have not shown any such toxicity. The injection procedure does carry risks of vitreous hemorrhage, retinal tear, retinal detachment, cataract formation, and endophthalmitis, but thankfully these complications are infrequent and are generic ones for an injectable agent. In a trial of repeated bevacizumab, ranibizumab, or aflibercept for the treatment of DME, conducted by Diabetic Retinopathy Clinical Research Network, 15–16 injections over 2 years were required on average to control edema (DRCR Protocol T, 2-year results). Drugs which are currently in clinical use for treating DME include VEGF inhibitors such as ranibizumab (Lucentis) and bevacizumab (Avastin) but there are few other options which include the soluble receptor for VEGF commonly known as the VEGF-Trap or aflibercept (Eylea) and intravitreal triamcinolone.

As discussed, because of the complexity and multifactorial nature of DR, there appears to be potential for discovering numerous biomarkers based on inflammatory molecules (like cytokines, chemokines and their respective receptors) or metabolites (such as homocysteine; Hcy) or various species of RNAs molecules (for example; miRNAs, circRNAs, ncRNAs, etc.) all stemming from inflamed retinal cells during DR disease pathogenesis. Anti-VEGF therapy in PDR is effective but requires indefinite treatment and for DME it is the mainstay of treatment, but approximately 40% patients in the DRCR Protocol I study, still had persistent edema at 6 months despite repeated injections. Furthermore, current treatments of anti-VEGF are inefficient as proven with the re-occurring of the edema within weeks or months of administration and thus repeated doses are invariably needed. Therefore, VEGF inhibition alone may not be enough and able to achieve neutralization of the inflammatory cascade and pyroptosis process that are responsible for BRB breakdown in the eyes. Thus, inhibition of inflammatory mediators could prove fruitful in DME therapies since cytokines and chemokines are upregulated in blood, aqueous and vitreous humor and these factors tend to accelerate DR pathogenesis. Other interventions or approaches are urgently needed to reverse the growth of retinal NV and treat DME (Cheung et al., 2010). A few newer versions of therapeutic strategies that can successfully target vascular complications in diabetes have also been developed such as the selective inhibition of sodium-glucose co-transporter 2 (SGLT2). These drugs have been recommended for the treatment of patients suffering from diabetes because of low risk of hypoglycemia and no weight gain. The principal mode of actions of the drugs rely upon the observations that up to 90% of the glucose is filtered by the kidney is absorbed back by a low-affinity/high-capacity SGLT2 that is expressed in the S1 and S2 segments of the proximal tubule in the kidneys. Thus, the blockade of SGLT2 promotes the urinary glucose excretion leading to the improvement in hyperglycemia in an insulin-independent manner. In fact, clinical studies proved that SGLT2 inhibitors reduce blood pressure, body weight, and serum uric acid levels and thus could also ameliorate cardiovascular risks in diabetics (Yamagishi and Matsui, 2016). It was proposed that patients with steroid-resistant DME, and those with refractory to the dipeptidyl peptidase-4 inhibitors (DDP4) the visual symptoms and macular edema might be treated by these SGLT2 inhibitors (Yoshizumi et al., 2018).

Also, DR treatment varies depending on the extent of the damage done by the disease itself. Some diabetics may require laser surgery to seal their leaking blood vessels or to even prevent blood vessels from leaking further while other might need immediate intravitreal injections to decrease inflammation or to reverse the course of NV. PDR patients may also need surgery to remove and replace a part of the vitreous or to repair TRDs. In the worst-case scenario, over time scar tissue in or on top of the retina can form which can cause retinal detachment and eventual cell death. While many of these detachments can be anatomically repaired with surgery, visual outcomes are mixed and frequently remain poor (**Figure 2**). DR can be diagnosed through a comprehensive eye examination. It is essential to know the patient’s history to determine vision difficulties because other co-morbidities may affect vision too. Therefore, visual acuity measurements are needed to determine how much central vision has been altered or lost. The doctor can also check refraction to determine whether a new eyeglass prescription would help the affected patient. Measurements of the intraocular pressure can also assist in deciding the appropriate treatment modalities for the diabetics. Supplemental testing may include retinal photography or optical coherence tomography (OCT) to analyze retinal layers’ integrity as well as to screen for macular edema (Bhaduri et al., 2017). Fluorescein angiography can readily identify NV, microaneurysms, and intra-retinal or intravitreal leakage (Takase et al., 2015; Hwang et al., 2016; Nesper et al., 2017). As detailed above, the treatment for DR depends on the stage of the disease, and the main aim of any treatment is to diminish or prevent disease progression. During the early stage of NPDR, strict monitoring is advised. Following physicians’ recommendation concerning diet and exercise coupled with blood sugar monitoring can help in following the disease progression. Lifestyle changes that promote physical activities such as regular exercise along with a healthy diet can potentially reduce blood glucose levels in patients suffering from T2D and reduce the risk of developing CVD and related pathologies. Physical activity increases insulin sensitivity and increases expression of the GLUT-4 transporter, which increases glucose uptake. Also, exercise tends to decrease blood MMP-9 levels in the diabetic patients (Kadoglou et al., 2010; Dehghan et al., 2016). Furthermore, because DR is typically diagnosed via a formal examination of the eyes by an expert in the clinic, this limits the number of patients that can be screened at a given time in a day. Therefore, an easily accessible and a reliable screening biomarker centered approach for timely DR detection (and prognosis) would also have tremendous practical value for disease management. This discovery could allow clinicians to identify susceptible patients early on that are in an urgent need of further assessment and/or treatment.

## Epigenetic Modification of the Genome in Diabetic Retinopathy

**FIGURE 3:**
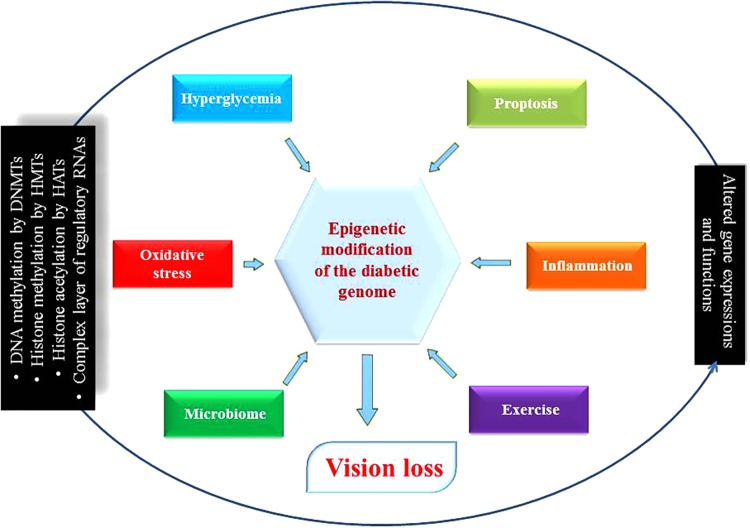
Diabetic genome modification. Different environmental determinants modulate genes’ expression and functions as a result of epigenetic modifications. Some are beneficial for health such as microbiome and exercise, but others are harmful like hyperglycemia, pyroptosis, inflammation and oxidative stress disrupting ocular physiology and homeostasis that can lead to diabetic retinopathy. DNMTs, DNA methyltransferases; HMTs, *histone* methyltransferases; HATs, histone acetyl transferases; circRNA, circular RNA.

Emerging evidence suggests that apart from one’s genetic makeup, the environment does affect DR phenotypic outcomes. Previous clinical trials have underscored the advantages of glucose control in preventing and diminishing DR-related complications, but studies have also demonstrated that strict control of glucose in diabetics does not suddenly stop DR progression (Mishra and Kowluru, 2016). In other words, the benefits arising from good control usually persist beyond the period of glycemic control. These specific observations hint at the importance and the existence of epigenetic regulatory mechanism(s) that have been revealed during the last few years. Recently, many types of epigenetic regulations, such as histone post-translational modifications in chromatin or DNA methylation, and the involvement of specific signature(s) governed by various RNA species have been associated with DR. Such discoveries point to the fact that the new field of “epigenomics” and its implications in DR and related pathologies are continually evolving such as the expression of pathological genes was found to be altered in endothelial and vascular smooth muscle cells without inducing any changes in the underlying DNA sequences of these cells (**Figure 3**). Further, besides the phenomenon of “metabolic memory” other events at play may determine the long-term epigenetics outcome(s) in cells and organs of the patients even after the underlying causes have been remedied (Zhang et al., 2012; Kowluru, 2017). Although some progress regarding the role of epigenetics in diabetes complications such as CVD has been made, the significance and importance of epigenetic mechanism(s) in DR remain mostly unknown (Pasquier et al., 2015). Researchers strongly believe that epigenetic modifications seem to play essential roles in the progression of DR (Zhong and Kowluru, 2011). Due to incredibly rich mechanistic pathway(s) that involves epigenetics (e.g., miRNAs’ expression, DNA methylation patterns, histone post-translational modifications, etc.), there exists a true potential for further research in DR to discover new details with potential therapeutic implications. We know that hyperglycemia has been shown to influence changes in the structure of chromatin through activating signaling pathways and hitherto unknown components (Qiu, 2006). In fact, many essential enzymes are specifically activated depends on the state (i.e., active or repressed) of chromatin. Equally, inflammatory genes in retinal cells that are regulated by epigenetics have also been demonstrated. Histone modifications by enzymes such as histone methyltransferases, histone demethylases, histone acetyltransferases, and histone deacetylases can reversibly change gene expression patterns (Pradhan et al., 2016; Zhang et al., 2017). Therefore, elucidating epigenetic regulation could certainly lead to the discovery and development of novel therapeutic agents for DR. Popularly known as ‘epi-drugs,’ such as histone demethylases, HATs, DNA methylation inhibitors, and HDACs are presently being analyzed for other medical conditions like cancer (Shi, 2007). It is hoped that available medicines could be used for their potential ability to alter epigenetic markers and thus also become an essential part of the therapeutic regimen for DR in the coming future (Zeng and Chen, 2014). As such, well-defined animal and cell based disease models with and without related interventions could lead us to understand the finer details of epigenetic regulations and how to employ them to prevent DR (Reddy et al., 2015). It is anticipated that the ongoing human epigenome research project will improve our understanding further of epigenetic states in normal health and the disease states (**Figure 3**) (Slomko et al., 2012).

Again, poor glycemic control and epigenomics have close relationship with DR as demonstrated in preclinical studies showing upregulation of HDAC1, HDAC2, and HDAC8 in the retinas of streptozotocin (STZ)-treated rats. Additionally, impairments in histone H3-specific acetyltransferase activity suggested that epigenetic metabolic memory might be a reason for continued disease progression even when blood glucose level has returned to normal (Zhong and Kowluru, 2010). Furthermore, antioxidants such as Sod2 (manganese superoxide dismutase parent gene) can prevent DR development in animals suggesting its role in DR. It is appropriate to mention that Sod2 is heavily regulated by epigenetic controls. Studies have demonstrated that epigenetic changes of retinal Sod2 play an essential role in the development of DR and the creation of traceable metabolic memory (Zhong and Kowluru, 2011, Kowluru, 2013). Also, hyperglycemia increases acetyl H3K9, H4K20me3 via NF-κB controlled genomic sequences encoding retinal Sod2, SUV420h2, and concurrently enhances the interactions between NF-κβ subunit of p65 to H4K20me3 and acetyl H3K9. Surprisingly, hyperglycemia reversal did not prevent these observations. SUV420h2; the methylation enzyme, is upregulated in retinal capillary cells and the retina during high glucose concentration. Furthermore, silencing of SUV420h2 prevents activation of H4K20me3 in Sod2 indicating the role of SUV420h2 in creating the metabolic memory in DR (Zhong and Kowluru, 2013). More specifically, hyperglycemia downregulated the signature methylation patterns (such as H3K4me1 and H3K4me2) and that can precisely be regulated by lysine-specific histone demethylase 1 (LSD-1) along with Sp1 interactions at the Sod2 promoter. It is known that LSD-1 demethylases (H3K4 and H3K9), and the subsequent methyl signature removal in H3K4 is associated with repression of the transcriptional profile. Interestingly, decreased H3K4me2 and increased LSD-1 at Sod2 also lead to the development of DR (**Figure 3**) (Forneris et al., 2008).

Matrix metalloproteinases play important roles not only in diabetic nephropathy and cardiac diseases but in retinopathy also as discussed earlier. Retinal MMP-9 is activated during diabetic damage to the eye initiating apoptosis of the capillary cells. During diabetes the promoter region of retinal MMP-9 exhibits an upregulation of acetyl H3K9, p65, LSD-1, and a downregulation of H3K9me2. Inhibition of LSD-1 via siRNA improves those changes and the impaired MMP-9 detrimental effects in DR (e.g., apoptosis) (Kowluru and Shan, 2017). From these observations and others, it appears that regulation of MMP-9 activities by epigenetic modifications as well as by genetic polymorphisms have important roles in the pathogenesis of DR as reviewed by Singh and Tyagi (Singh and Tyagi, 2017c). Also, the aberrant DNA methylation patterns of CpG dinucleotides in certain genetic elements are fundamental mechanisms in the regulation of gene expression status, thus disrupting the standard transcriptional regulatory machinery of a cell irrespective of a disease state (Micevic et al., 2017). Overexpression of methylation of the CpG dinucleotides in the polymerase-γ region affects mtDNA binding compromising the activity of transcription because replication enzyme remains downregulated along with mtDNA ‘D’ loop region damage. These changes were not reversed in the absence of high blood glucose level (Tewari et al., 2012).

## The Micrornas and Their Role(S) in the Development of Diabetic Retinopathy

The expression levels of miRNAs are also affected during DR development. For instance, miRNA-200b is significantly upregulated in T1D model of DR. Moreover, a mimic of miR-200b decreased oxidase resistance 1-impaired oxidative stress progression during transfection. STZ-treated rats exhibited changes in the expression of 37 miRNAs in DR: upregulation of miRNA-96, 124, 182, 183, 204, and 211 while miRNA-10a, 10b, 199a-3, 219-2-3p, 144, and 338 were downregulated (Wu et al., 2012; Murray et al., 2013). Other studies have also shown an increase in the expression of miRNAs (miRNA-21, 132, 146, 155) that are responsive to NF-κB in RECs. The micro-RNAs (miRNA-17-5p, 18a, 20a, 21, 31,133) which are regulated by VEGF are also upregulated in RECs and retinas. As widely known that NF-κB is a crucial regulatory molecule in our immune system and is associated with DR development wherein it induces apoptosis in retinal pericytes. The miRNAs (miRNA-21, 146, and 155) that may be regulated via NF-κB were also upregulated in diabetic RECs indicating that NF-κB can activate miRNA-146 expression. The higher expression of miRNA-146 inhibits activation of interleukin-1β (activation-induced NF-κB) in RECs. Therefore, enhanced expression of miRNA-146 could be influential in controlling DR progression (Kowluru and Koppolu, 2002; Brooks et al., 2004; Taganov et al., 2006; Gatto et al., 2008; Sheedy et al., 2010; Kovacs et al., 2011). Increased expression of VEGF, and the miRNAs that respond to p-53 indicate the association of miRNAs in the pro-angiogenic or pro-apoptotic role(s) of VEGF in DR. Downregulation of miRNA-200b and upregulation of VEGF has been observed in human umbilical vein endothelial cells (HUVECs) and RECs. The miRNA mimic treatments *in vitro* in endothelial cells and *in vivo* (intravitreal injections) might counter the VEGF upregulation in DR. In animal models, miR-200b mimics intravitreal injections that were shown to be downregulated by VEGF-A (Long et al., 2010; McArthur et al., 2011; Li et al., 2017).

## Perturbation of the Redox System in DR

**FIGURE 4:**
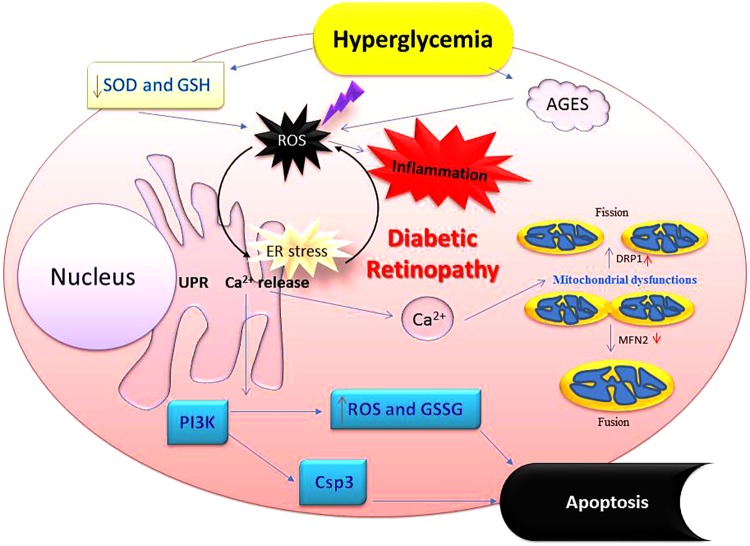
Perturbation of cellular redox by hyperglycemia promotes oxidative stress-inflammatory axis in diabetic eyes. Imbalance in redox control because of high glucose affects many aspects of ocular physiology including mitochondrial damage and synthesis of higher amounts of advanced glycation end products (AGES) leading to oxidative stress followed by the inflammation. These alterations deplete cells’ abilities to fight off physiological imbalance via antioxidants and glutathione. Oxidative stress also upregulates NADPH oxidase which increases production of various reactive oxygen species (ROS). Apoptosis is also accompanied leading to the loss of retinal cells. Csp3, caspase 3; GSSG, oxidized glutathione (glutathione-*s*-*s*-glutathione); GSH, glutathione (a tripeptide-λ-glutamyl-cysteinylglycine); NADPH, nicotinamide adenine dinucleotide phosphate hydrogen; SOD, superoxide dismutase; PI3K, phosphatidylinositol-4,5-bisphosphate 3-kinase.

Diabetes creates an excess of oxidants at the cellular level, i.e., oxidative stress. Many investigations have indicated a direct association between inflammation, oxidative stress, and diabetes during DR development. This relationship may help explain the harmful effects of inflammation and pyroptosis in both the retinal vasculature and retina and the consequent abnormalities in visual function. Oxygen consumption by mammalian cells is essential for sustaining aerobic life and the standard homeostatic mechanisms. This process is predicated upon a strict control in the level of reactive oxygen species (ROS) production and their removal from almost every tissue and cell type in our body. Although the initiators of oxidative stress in DR are not well understood, it is agreed that oxidative stress is influential in DR (**Figure 4**). Antioxidants have been shown to alter the course of retinopathy development in animal models (Tanito et al., 2005; Rosales et al., 2010; Kumar et al., 2012; Kiang et al., 2014; Arellano-Buendia et al., 2016), but the results obtained from limited clinical trials have not demonstrated the same effects (Berman and Gombos, 1989; Chiarelli et al., 2004; Garcia-Medina et al., 2011). However, antioxidants are routinely prescribed for a range of other chronic disease conditions. Thus, more evidence-based clinical trials are needed to justify therapeutic effects of antioxidants in the development and progression of DR (Kowluru and Chan, 2007).

Reactive oxygen species such as superoxides are potent secretagogues for cytokines secretion. They can promote inflammation through various pathways that can serve as a key pathogenetic event that can damage vascular cells and thus increasing the vascular permeability. Cytokines and chemokines released into the retina become potent recruiters of neutrophils and a host of other cells (De Groef et al., 2015; Liu et al., 2016). ROS induce NF-κB activation which drives an inflammatory response via increasing the levels of nitric oxide (NO), prostaglandins, and other cytokines (Schreck et al., 1992). The level of interleukins significantly increased in the vitreous of PDR patients, and the retina of experimental animal models such as diabetic rats and mice, particularly IL-1β, IL-6, and IL-8 are (Yuuki et al., 2001). This study showed that IL-1β increased significantly in retinal capillary cells under hyperglycemic conditions (Carmo et al., 2000; Kowluru and Odenbach, 2004b). Interestingly, IL-1 activation can lead to release of more ROS and NF-κB translocation to the nucleus, thus creating a continuous inflammatory response (**Figure 1**) (Chang and LoCicero, 2004; Koenig et al., 2016). Further, IL-1β is associated with apoptosis of retinal capillary cells, probably via NF-κB and caspase-3 activation (Kowluru and Odenbach, 2004a; Gupta et al., 2017; Mendiola and Cardona, 2017). IL-1 could upregulate prostaglandin E_2_ (PGE_2_) via the catalytic enzyme cyclooxygenase-2 (COX-2). Both are associated with DR development by modulating vascular permeability and angiogenesis through VEGF (Ozawa et al., 2011).

Oxidative stress has also been linked to chronic inflammation leading to the changes in messages that are encoded by stress response genes (Reuter et al., 2010). These signals, in turn, induce diabetic complications such as retinopathy. It is worth noting here that retina contains relatively higher levels of polyunsaturated fatty acids (PUFA) and uses more oxygen by mass than any other part in the human body. It also exhibits higher glucose oxidation than any other tissue in mammals. Various species of ROS (e.g., superoxide, hydrogen peroxide, etc.) are elevated in retinal tissues of diabetic rats while the activities of the enzymes that specifically quench these ROS such as superoxide dismutase, catalase, glutathione reductase and peroxidase are decreased in the diabetic retinal cells. Prominent antioxidants such as beta-carotene, vitamin C, and E, and GSH are decreased likewise in the diabetic patients. Biochemically, glucose phosphorylation results in fructose 3-phosphate which further breaks down into three deoxyglucoses. Both are glycation agents which can then enter the advanced glycation pathway as oxidative stress activates signals that can lead to the generation of AGE products, the induction of the hexosamine biosynthesis, enhanced generation of insulin-like growth factor 1 (IGF-1), and, most importantly, activation of the polyol pathway (**Figure 4**) (Singh et al., 2015). Glucose is reduced to sorbitol via AR which is then converted to fructose by sorbitol dehydrogenase (SDH) in the polyol pathway. AR and SDH are two primary enzymes that are heavily used in the polyol pathway. The conversion of glucose to sorbitol requires abundant NADPH. This competes with the reaction that forms the antioxidant GSH, which also requires NADPH. The use of NAD^+^ may also create an imbalance in the overall ratio of NADH/NAD^+^, the excess NADH could become a substrate for NADH oxidase, which in turn, can generate more oxidative species in the cells. Many retinal cell types contain AR, including ganglion cells, Müller cells, endothelial, pericytes, as well as retinal pigment epithelial (RPE) cells. They all are subjected to polyol pathway metabolism in diabetic eyes. Also, retinal cells in diabetic animals show an increase in the levels of sorbitol, fructose, and oxidative stress, and these could be prevented through compounds that can specifically inhibit the biological activity of the AR enzyme (Sato et al., 1993; Lorenzi, 2007). AR can potentially deplete the levels of NADPH in the polyol pathway thereby lowering its availability for glutathione reductase, an important antioxidant. In other words, the overall ability of a cell to respond to oxidative stress decreases significantly under hyperglycemic conditions (Lorenzi, 2007).

## Role of Signaling Pathways During Diabetic Retinopathy

Obesity and T2D are associated with galectin-3 (Gal-3) upregulation which is known to mediate inflammation and clearance of glucose adducts. Gal-3 knock-out (KO) mice were shown to upregulate adipose and inflammation in systemic tissues. These animals also exhibited impaired fasting glucose levels, increased response to glucose tolerance test (GTT) along with decreased expressions of adiponectin, GAL-12, ATGL, and PPARγ. Impaired glucose metabolism can precede the development of excess fat deposition thereby leading to the systemic inflammation. These observations indicate the essential role of Gal-3 in adiposity, inflammation, and glucose metabolism (Pang et al., 2013). Further, Gal-3 has been suggested as an obvious therapeutic target for altering degeneration of the retina due to its role in causing hypoperfusion. Additionally, the H-Ras-mediated signaling cascade has been shown to activate MMP-9 in RECs in humans and in animal models. This suggests that Ras-ERK pathway plays a vital role in MMP-9 activation in the retina. In fact, the sustained activation of this pathway can result in capillary cells apoptosis in retina. Thus, MMP-9 may present a plausible therapeutic target (Mohammad and Kowluru, 2012). The biology of visual processing has been linked to NO signaling pathway and changes in this pathway have been noticed during diabetes. Interestingly, upregulation of neuronal nitric oxide synthase (nNOS) occurs via adrenomedullin (ADM) in this pathway. Diabetic individuals exhibit an upregulation of ADM in their eyes. Inhibiting ADM/NO signaling pathway in DR might downregulate NO in the retinal cultures treated with high glucose. Ruboxistaurin (PKC β inhibitor) downregulates ADM expression and activity, and electroretinography (ERG) showed that this change helped preserve the retinal functions suggesting that ADM/NO pathway could also serve as a potential therapeutic target for DR (Midena and Pilotto, 2017).

## Discussion

Blindness constitutes a significant global health concern and has a powerful impact not only on afflicted patients but also on their families. It carries an enormous socio-economic burden on the society as well. Blindness is further augmented by an increasing incidence of diabetes cases and DR in particular because DM patients are living longer than ever before (Tabish, 2007; Zheng et al., 2012; Abuyassin and Laher, 2016; Leasher et al., 2016). The most threatening complications of DR are DME and PDR. To address this public health menace, effective strategies must be devised to control severe visual disability by implementing appropriate screening tools for early detection by devising standard operating procedures (SOPs) that should essentially include: (1) DR prevention, (2) reinforcing DR treatments, (3) using appropriate technologies to diagnose and treat early, and (4) increasing awareness about DR prevention methods (Romero-Aroca et al., 2010; Khandekar, 2012; Mi et al., 2014).

**FIGURE 5:**
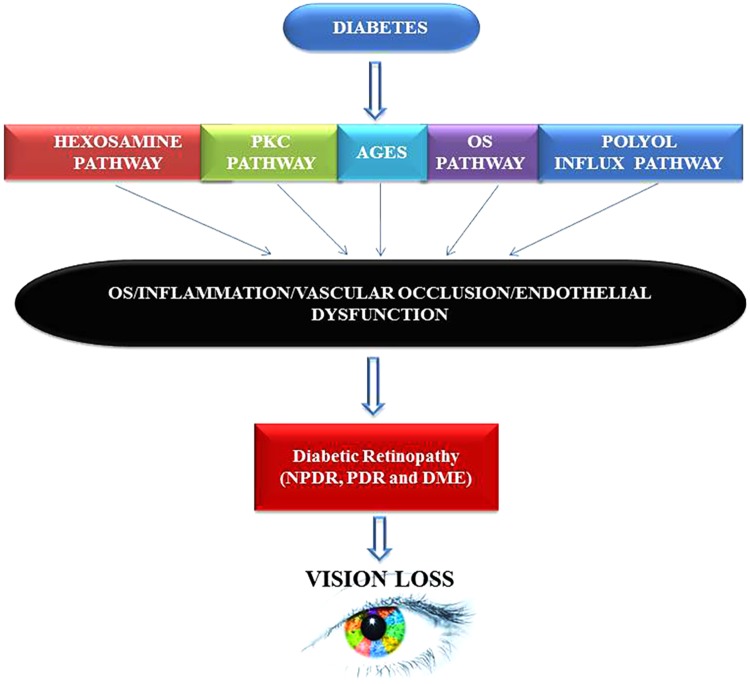
Hyperglycemia inflicts damage leads to neuropathy, diabetic retinopathy (DR), and vision loss via multiple mechanisms. Chronic hyperglycemic environment is a powerful inducer of proliferative diabetic retinopathy (PDR) and diabetic macular edema (DME) phenotypes in humans. Both PDR and DME can cause mild to severe vision impairment in patients that can result into complete blindness if timely intervention is not sought. PKC, protein Kinase ‘C’; AGES, advance glycation end products; OS, oxidative stress.

By presenting numerous pieces of evidence both at the cellular and molecular levels, we have tried to combine together an explanation that may enlighten future paths toward understanding and developing appropriate DR prognostics, diagnostics and therapeutics tool-kits that might help researchers and clinicians to make sound decisions regarding diagnosis, treatment, and future research. Taking cues from other chronic medical conditions such as cancer, Alzheimer, Parkinson or CVD, it has recently been discovered that inflammation is strongly associated with DR. Even the processes of aging and hormonal abnormalities are also being considered from an inflammatory perspective or at least related to inflammatory responses in our body (Joussen et al., 2004; Wellen and Hotamisligil, 2005; Sjoholm and Nystrom, 2006; Sears and Ricordi, 2011). Early detection is the key and can undoubtedly help institute appropriate management practices for patients with available options that may still prevent, delay, or mitigate vision loss from DR. The primary mechanisms that may explain DR pathology include formation of AGEs, the polyol pathway (**Figure 5**), oxidative stress, epigenetic modifications, inflammation, and pyroptosis (Safi et al., 2014). However, the main underlying factors that are linked directly with diabetes remain hyperglycemia, oxidative stress, increased production and synthesis of growth factors, and the activation and secretion of a host of inflammatory molecules that in principle can deregulate retinal physiology. AGE formation can lead to an imbalance in growth factors and inflammation imbalances in the ocular compartment of diabetic patients (Obrosova and Kador, 2011). Further, upregulation of polyol via the hexosamine pathway is also associated with DR (Sharma et al., 2005; Kowluru and Chan, 2007; Safi et al., 2014; Singh et al., 2017).

Vision problems arising from DR become evident in later stages of the disease but molecular changes that are operate and regulate the disease process in general and in the retina in particular start quite early when patients are still at the pre-diabetic stage. Thus, efforts should be focused on seeking new and robust ways to detect DR at an earlier time point before a patient presents with an advanced disease. To do this, work should also be directed to harness the power of the latest ‘omics’ technologies including the refinement of currently available imaging tools for detecting subtle early types of changes in the retina and its elegant vascular architectural network. New evidence reflects that epigenomics affects gene expression in diabetes, confirming the metabolic memory concept, although much remains to be discovered in this evolving space. Interestingly, epigenomics can also aid in determining the interactive role, if any, played by genetic variants that stand to protect or promote diabetes susceptibility and disease progression in various ethnicities and races around the globe (Singh and Tyagi, 2017c). Also, it will be worthwhile to assess whether the combination of one’s lifestyle such as exercise coupled with a healthy diet can help reduce complications by altering the epigenetics profile of the ocular cells. In that context, numerous investigations have demonstrated the benefits of using marker(s) associated with diabetes (Ronn and Ling, 2013). Current understanding espouses that epigenetic changes are reversible, and some therapies are under development to harness this feature with drugs that are being touted as “epi-drugs” and antagomirs (inhibitors of miRNA molecules) which might someday be used to complement existing treatment options (Baylin and Jones, 2011; Kato and Natarajan, 2014). As outlined earlier, inflammation appears to be a driver of complications due to diabetes. Therefore, variations in epigenetics could be analyzed in circulating inflammatory cells (e.g., monocytes) (Reddy et al., 2015).

Finally, the standard of clinical care for DR has evolved dramatically in the last 10 years. For decades, laser photocoagulation has been the first-line therapy for both PDR and DME (Evans et al., 2014). However, at present, intravitreal anti-VEGF injections are the standard for DME and their effectiveness has been demonstrated in clinical trials. They are also easy to administer and provide rapid improvement in vision. Anti-VEGF therapy also provides an alternative to panretinal photocoagulation for the treatment of PDR. DRCR.net protocol S trial revealed that intravitreal ranibizumab (Lucentis) was non-inferior to pan-retinal laser photocoagulation in treating PDR. All of this has given confidence to ophthalmologists to prefer anti-VEGF injections over the laser intervention for DME, which has essentially become a second-line therapy for this indication. However, most agree that anti-VEGF agents are far from perfect as they are short-acting, relatively costly, and carry the risks of endophthalmitis and stroke, which are not concerns with retinal laser photocoagulation. A clinical trial performed by Diabetic Retinopathy Clinical Research Network (DRCR.net) protocol T found that almost half of patients with DME required additional laser treatment after 6 months of anti-VEGF therapy.

## Conclusion

Globally, diabetes incidence is rising. Currently, therapeutic methods are unable to restore vision in advanced cases, and thus there is an unmet need for innovating newer approaches to tackle important cellular processes like oxidative stress, inhibition of signal transduction pathways, inflammation and pyroptosis (e.g., the protein kinase C cascade and AR pathways). Future treatments should be able to target different molecules that are vital to DR development (e.g., hepatocyte growth factors, MMP-9). Finally, novel mechanisms of therapeutic delivery should also occupy a front seat to treat and manage people suffering from this seemingly unstoppable health crisis (Dedania and Bakri, 2015). It appears that the mechanisms, events and pathways that we covered in this manuscript seem to act in conjunction in several ways. Since epigenetics is still a nascent discipline, we should be cautious not to draw hasty conclusions. Impact of the high prevalence of diabetes on people’s health and the economic burden that it brings with it deserve a fresh look. We see such an intervention as being crucial to reduce the socio-economic burden of this incurable malady on global economy.

## Author Contributions

All authors participated and contributed intellectually toward collecting literature pertaining to inflammation, pyroptosis and diabetes along with writing, reviewing and editing the manuscript before submission for its publication.

## Conflict of Interest Statement

The authors declare that the research was conducted in the absence of any commercial or financial relationships that could be construed as a potential conflict of interest.

## Abbreviations

AR: aldose reductase
BRB: blood-retinal barrier
CDC: center for disease control
CVDs: cardiovascular diseases
DM: diabetes mellitus
DME: diabetic macular edema
DR: diabetic retinopathy
ECM: extracellular matrix
HbA1C: hemoglobin A1C
Hcy: homocysteine
HNE: 4-hydroxynonenal
MI: myocardial infarction
MMPs: matrix metalloproteinases
NV: neovascularization
PDR: proliferative diabetic retinopathy
PRP: pan-retinal photocoagulation
PUFAs: poly unsaturated fatty acids
RPE: retinal pigment epithelium
T1D: type I diabetes
T2D: type II diabetes
TGF-β: transforming growth factor-β
TRD: tractional retinal detachment
VEGF: vascular endothelial growth factor
WHO: World Health Organization
